# Experimental and Numerical Assessment of Iso-Flux Cooling with Low Reynolds Pulsating Water Flow

**DOI:** 10.3390/s22197487

**Published:** 2022-10-02

**Authors:** Ferenc Szodrai

**Affiliations:** Department of Building Services and Building Engineering, Faculty of Engineering, University of Debrecen, 4028 Debrecen, Hungary; szodrai@eng.unideb.hu

**Keywords:** low Reynolds, laminar, pulsating, solenoid, thermal analysis, iso-flux, large-eddy simulation, water flow

## Abstract

Almost every scale in thermal engineering requires performance optimization to lessen energy demand. The possibility of using pulsating flow for water cooling was investigated both experimentally and numerically. The experiments were conducted below a 60 mL∙min^−1^ flow rate and frequencies of 3.3, 4, 5, 6.6, and 10 Hz. The flow rate and temperatures were monitored while the solenoid valve was actuated and cooled with thermoelectric coolers. The measurements were replicated by using commercially available software capable of doing large-eddy simulations with coupled thermal modelling. Thermal boundaries were created by using steady inflow temperature and iso-flux conditions. The experimental and numerical results were compared and evaluated. The results show that the Nusselt number of the examined pulsating flow was lower when compared to constant flow scenarios at the corresponding averaged flow rate.

## 1. Introduction

Most thermal systems operate with a steady flow. Numerous investigations [1,2] have demonstrated that disrupting both the velocity and temperature boundary layers enhances the extractable heat. Amplification of heat transfer in pipes can be achieved by having wavy walls [3,4,5] or by swirling the flow [6]. Yet, as Shahzad Nazir et al. [1] in a comprehensive review concluded, often a smooth tube can cause more favourable heat transfer enhancement than various types of corrugation in pipes.

A piston pump may produce two forms of unstable flow: oscillating and pulsating. The former has limited application and potential to improve heat transfer [7,8]. The latter has a primary flow direction, which may result in comparable positive thermal increases [9,10]. Pulsating flow has a wide range of applications, including Stirling engines [11], geothermic probes [12], or can occur in blood flow [13]. Another use of pulsating flow is when the flow rate is limited, such as in hardware cooling or surface cooling in buildings.

A well-known way of describing the temperature change of a cooling object is the lumped heat capacitance model. However, when a heat load (heating or cooling) is present in an active system, both the fluid temperature and the surface temperature alter at the beginning of the process. As a result, the isothermal boundary condition is unsuitable for analysis. To represent the temperature of the body along the flow, an iso-flux boundary condition is needed.

To model these kinds of scenarios, the large-eddy simulation (LES) method can be used, which often produces satisfactory results for iso-flux models, as demonstrated by the work of Ould-Rouiss et al. [14] and Holgate et al. [15].

Experimental and numerical investigations have been carried out in recent years.

Basiak et al. [16] investigated two-phase flow, with an emphasis on velocity visualization and assessment. While Guo et al. [17] performed thermal analysis, it was only for one frequency at a high flow rate.

The novelty of this study is that it focuses exclusively on low Reynolds water flow as well as its actuation with the combination of precise temperature and flow measurement and the LES method along with the comparison with the iso-flux theoretical model. A great deal of research [4,18,19,20] emphasised the relationship between pulsating flow and heat transfer, although Reynolds number (Re) was above 2000. This paper presents such experimental research which is considered to be uncommon [21]. The assessments concentrate on changes in both flow and temperature. Thermoelectric devices were employed for iso-flux cooling load; however, the study did not look at how they performed. The specifications are only mentioned for the repeatability of the measurement.

## 2. Materials and Methods

To measure the cooling effect of pulsating flow, the experiments were performed first, followed by the numerical analysis. Models of computational fluid dynamics (CFD) were compared with experimental data. The outcomes of numerical models and experiments were compared and evaluated.

### 2.1. Experimental Setup

The experimental measurements were aimed to find an optimum case where the pulsating flow becomes superior to the steady flow. In addition, experimental results were used for the validation of the numerical models. The measuring bench was a combination of water and electric circuits with a heat sink at the intersections. For the analysis, a water medium was used, which was cooled by two thermoelectric heat pumps (TEC; type: TEC1-12706).

**Figure 1:**
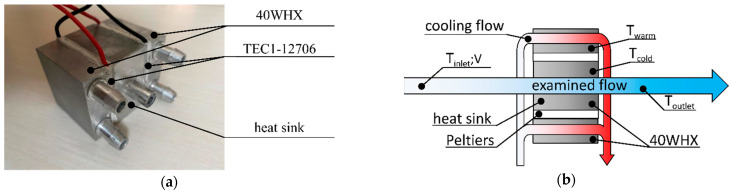
The assembled heat sink (**a**) and the scheme of the heat sink measurement (**b**).

**Figure 2:**
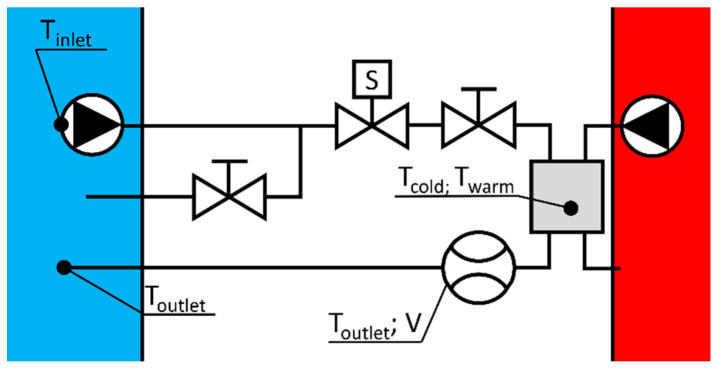
Scheme of the water circuits (reservoir of the examined circuit: blue; cooling circuit: red).

Figure 1a depicts the geometry of the aluminium heat sink. It was produced for this measurement. The scope of the experimental measurements was the “examined flow”, which crossed the centre of the heat sink (see Figure 1b). The cold sides of TEC were symmetrically attached to the 40 mm by 40 mm flat surfaces of the heat sink. The cold (T_cold_) and warm side (T_warm_) temperatures of the Peltiers were monitored to calculate the cooling power demand (${\dot{Q}}_{c}$). The T_cold_ thermometer was installed in the heat sink, whilst the T_warm_ thermometer measured the surface temperature of the aluminium water heat exchanger (40WHX). The TEC device voltage (U) and total electric circuit power consumption (Ṗ) were monitored. The T_warm_, U, and Ṗ were only monitored to ensure the stable operation of the measurement. To verify these observations, a thermal camera was utilised to measure the temperatures of the ceramic plates of the Peltiers. The water used in the experiment was pumped from a reservoir tank. The volume of the reservoir was large enough (20 litres) to have no cooling interference (T_outlet_) caused by the return flow. The flow meter’s built-in temperature sensor, and another temperature sensor right before the water flowed into the reservoir, were used to measure the T_outlet_ (see Figure 2).

Excess heat from the TEC devices had to be removed by cooling the flow through the type 40WHX heat exchangers (see Figure 2). This flow rate and the temperature of the cooling circuit were not monitored. Two 5 W pumps ensured the examined and cooling flows, with the magnitude of the examined flow adjusted via a throttle valve. Table 1 shows the measuring devices and parameters that were used.

The flow rate (V) of the examined flow was limited by the measuring range of the flow meter (60 mL∙min^−1^), which became the experimental limitation. The sampling interval of $\dot{V}$ was 5 ms, while the temperatures (T_cold_; T_warm_; T_inlet_; and T_outlet_) were 10 s. It is worth noting that the type SLF3-1300F flow meter measures the flow based on the fact that it adds heat to the water, thus temperature is measured; however, the device is only calibrated for flow measurements and not temperature. Temperature data will be showcased, but only in normalised form.

A rapidly closing-opening solenoid valve (type: AIRVALVE-12VSS) was used to create the pulsating flow. A type “Arduino nano” microcontroller controlled the solenoid valve. The interval between opening and closing was half a cycle. The frequency of the entire cycle was used to differentiate the various cases. A T junction was installed in front of the actuator to reduce the water hammer effect caused by the sudden closing. The T-valve also ensured that the system pressure level should not rise by throttling the flow. It was assumed that the hydraulic resistance would be significant, so the flow would bypass the investigated area. As a result, the throttle valve was installed in the bypass branch to ensure the examined flow.

### 2.2. Simulation Setup

**Figure 3:**
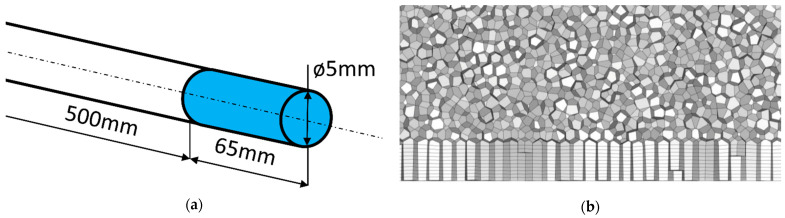
Domain geometry (**a**) and meshing (**b**).

The central hole of the heat sink was recreated in the simulations resulting in a 5-mm-diameter and 65-mm-long cylinder (see Figure 3a, a blue cylinder). The heat load was applied to the shroud, while the top and bottom served as inlet and outlet boundary conditions. Because the pulsating flow alters the inlet velocity profile, an additional domain was added to develop the inlet velocity profile rather than using a velocity profile function. This additional domain was one hundred times the diameter in length. In the flow development section, the shroud was adiabatic. Because of the high quality of the milling of the pipe walls, no-slip conditions were used.

Meshing was created to satisfy the y^+^ = 1 condition for Re = 5500, which was the maximum flow rate in the simulation series. The mesh structure was constructed of ten prismatic layers applied on top of polyhedral cells. Polyhedral cells were chosen over tetrahedral ones because they better represent low-velocity fluctuations [22]. Symmetry conditions were not used to examine the fully developed flow structure.

Mesh independence analysis was performed, three meshes were evaluated, and the scaling factor of the grid was two. The pipe outlet temperature was chosen as the control parameter. The three meshes showed minor differences in the grid convergence index (GCI) [23] calculation (see Table 2), thus simulations were carried out on the medium mesh. The averaged quality indicators of the medium mesh were aspect ratio 3.614, skewness 0.014, and orthogonal quality 0.986.

The wall-adapting local eddy-viscosity large-eddy simulation (WALE-LES) [24] method was used for the turbulence model. In the work of Renze et al. [25], this model demonstrated insignificant differences between experimental and simulated heat transfer results in an unsteady state. The inlet flow temperature (T_inlet_) and heat load (${\dot{Q}}_{c}$) of the heat sink shroud were constant in terms of boundary conditions. The iso-flux condition was used to observe the heat transfer process solely in terms of flow rate, while temperatures in the domain could change since temperature fluctuation of the incoming flow has a low influence on the heat transfer enhancement [26].

The model was isothermal, and the semi-implicit method for pressure-linked equations-consistent pressure–velocity coupling scheme was used for velocity and pressure-based solvers. Furthermore, PRESTO! was used for pressure discretisation, and bounded central differencing was used for momentum and transient formulations. For velocity 10^−3^ and energy 10^−6^, the magnitude of the convergence criterion was set.

The simulations were run in three sets. First, only the heat sink domain was modelled, with no inflow in order to assess temperature change over time. Then, there were scenarios with a steady flow. These were the benchmark cases for the pulsating flow rate cases, which were the subjects of the third set of simulations.

#### 2.2.1. Flow-Less Setup

The flow-less setup consisted of two pressure outlet boundaries and shroud cooling. The experimental conditions were used for the outlet boundary condition, which was 1 atm total pressure. The cooling power was 5, 10, and 20 W, and the initial temperatures were set to 10 and 20 degrees Celsius, respectively. The surface-averaged temperature of the heat sink (T_S_) and the volume-averaged water temperature (T_F_) were monitored. The timestep was 5 ms, and the total simulation time was 20 s.

#### 2.2.2. Steady Flow Setup

Each simulation set inherited the previous setups, but some additional modifications were implemented. As a result, a flow development zone was added to the steady flow. The constant flow cases focused on the low Re = 100–400 laminar regime (since the experiment was carried out in this range). The inlet boundary condition was a constant velocity function determined by the Re number (see Equation (1)).

(1) vinlet=Re·μ·D·ρ−1

where µ is the dynamic viscosity (0.001003 Pa∙s), D is the diameter of the pipe (5 mm), ρ is the density of the water (998.2 kg∙m^−^^3^). In each case, the initial flow rate was zero. To compare the cooling and heating effect, 20 W of heat load was also added to the list of simulations.

#### 2.2.3. Pulsating Flow Setup

Unsteadiness can be caused by non-iso-flux surfaces, created by thermal cycling of the heat source [27], yet iso-flux boundary conditions combined with LES usually show strong connections with experimental results [14,24]. The pulsating waveform could be of any shape (for example, sawtooth, square, or triangular) [28], but it performs best when it characterises a sinusoidal form, that is why a sinusoidal flow rate was set as the input boundary condition, as defined by Equation (2).

(2) Re=Re¯·1+β·sinτ·f

where Re¯ represents the time-averaged mean Re number, β is the amplitude, τ the flow time, and f is the frequency of the wave cycle. Flows were examined in a wide range of frequencies (3.3; 4; 5; 6.6 and 10 Hz) at a constant amplitude of 1 and with 5 W of cooling load. The simulations were carried out with Ansys Fluent 2022 R1. (ANSYS Inc., Canonsburg, PA, USA)

## 3. Results

### 3.1. Measurement

The measurement results show that the reservoir volume was large enough to minimise the cooling effect, with the total deviation (decrease) in reservoir temperature being less than 0.2 °C. The cooling circuit designed to remove heat from the warm side of the Peltier modules performed poorly because the heat load was much larger. When the temperature of the warm reservoir reached 45 °C, the measurement was stopped and the water in the cooling reservoir was replenished. This revealed a significant difference in cooling performance when the sets of measurements were examined separately. A steady flow rate and pulsating flow cycles were included in a set of measurements. Each case was measured for at least 5 min. To avoid any correlation between the increase and decrease of the frequencies, the order of the actuation was chosen at random.

#### 3.1.1. Flow Rate

When pulsation was added to a constant flow circuit, the time-averaged flow rate decreased. Pulsations can be generated by a piston [7] or a control valve. For this study, a solenoid valve was chosen with a response time of less than 50 ms. It was assumed that the flow rate should be constant at any frequency if the actuation of the valve were shorter than the actual cycle. Since the actuation was around 50 ms the largest examined pulsation frequency became 10 Hz.

**Figure 4:**
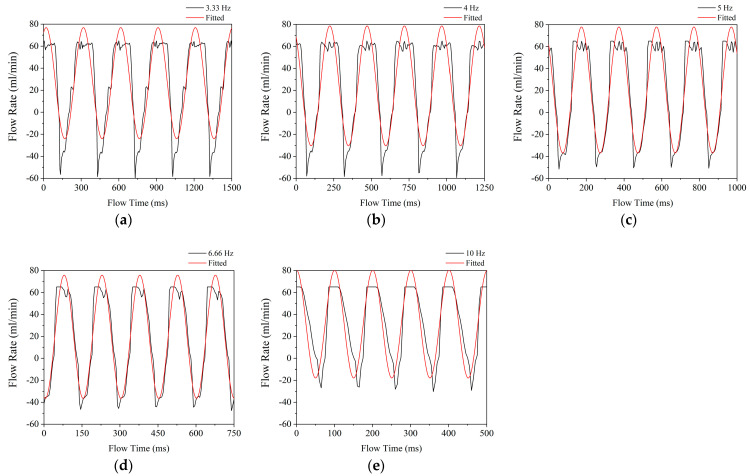
Sample of the measured flow patterns in the function of time at 3.33 Hz (**a**), 4 Hz (**b**), 5 Hz (**c**), 6.66 Hz (**d**), 10 Hz (**e**).

Five of the pulsating flows’ (3.3; 4; 5; 6.6 and 10 Hz) flow rates and time curves are presented in Figure 4. Additional sinusoidal curve fit was also added to determine the quality of the generated flow. The sinusoidal line can be expressed with Equation (3).

(3) $$\dot{V}=\bar{\dot{V}}+\beta_{V}\cdot \sin\left(\tau \cdot f+\varphi \right)$$

where $\bar{\dot{V}}$ represents the average flow rate, β_V_ denotes the flow amplitude, τ the flow time, f the frequency, and φ the phase angle. Table 3 shows the corresponding values.

Figure 4 shows that the actual flow curves are distorted at both the minima and maxima. Maxima distortions are caused by sudden openings and a delay before closing. Due to the limitation of the flow meter, flow rates exceeding 60 mL∙min^−1^ were not recorded. Despite the fact that a sinusoidal curve fit reveals that the maxima can be as high as 80 mL∙min^−1^. These distortions are straight lines at 10 Hz (see Figure 4e). When the frequency decreases, a slight fluctuation appears, which can be attributed to measurement inaccuracy. The distortion of minima indicates that there are some additional backflows present in the heat sink. The measured backflow could be due to the fact that the shock from sudden closing is only damped in front of the solenoid valve and not after it. The backflow indication can be attributed to the flexible piping because the valve was closed. The piping was made of a soft silicone hose, and it is assumed that the inertia of the water from the rapid velocity change contracted and expanded the hose, resulting in negative flow rate values.

Minima were peaking values; following a sudden drop, the flow rate reached the lowest point, followed by a rapid rise. Figure 4a,b reveal that at lower frequencies (3.33 Hz and 4 Hz) the minimum peaks at −60 mL∙min^−1^ and exponential rise up to about −40 mL∙min^−1^ then a slower increase begins. This pattern is also visible at a smaller scale at higher frequencies (see Figure 4c,d). Figure 4 additionally depicts that the steepness of the sudden drop reduces as the frequency rises, while the increase in flow rate remains nearly uniform.

It can be assumed that because the actuator valve was closed for a shorter period of time, it could not reach the minima. Therefore, the waveform became closer to the sinusoidal. However, during longer cycle periods, the flow profile develops. In addition, a rapidly closing valve at lower frequencies, on the other hand, produces a square [28] flow profile, which is undesirable.

The fitting of the measured values shows a good regression value (R^2^ = 0.807–0.923), thus it can be considered a sinusoidal flow. The 10 Hz scenario was the closest to the non-actuated 30 mL∙min^−1^ average flow rate. In the other cases, it decreased. This trend is also visible in Table 4, which displays the mean measured values along with the standard deviation and relative change compared with the 5 Hz case.

Lowering the frequency from 10 Hz resulted in a 51% decrease in mean flow rate at 6.6 Hz, which decreased by 2% at 5 Hz. A further decrease in frequency results in an increase of 4 mL∙min^−1^ (20%). This can be attributed to minima distortion.

#### 3.1.2. Temperature

To conserve energy, it should be assumed that as the flow rate decreases during cooling, the temperature difference increases. This would imply that the case with the lowest mean flow rate (6.6 Hz) should have the lowest outlet temperature. The water temperatures were monitored at the supply (T_inlet_) and return point (T_outlet_) of the reservoir. The 10 s averaged values are presented in Table 5. Repeated measurements with the KRV-200 sensors show that the 5 Hz case had significantly lower values.

To pick the most favourable scenario, both the average flow rate and the temperature have to be assessed by calculating the extracted heat from the water flow.

(4) $${\dot{Q}}_{c}=c\cdot \bar{\dot{V}}\cdot \rho \cdot \left(T_{\mathrm{outlet}}-T_{\mathrm{inlet}}\right)\;$$

where c was the specific heat of the water (4180 J∙kg^−^^1^∙K^−^^1^), T_inlet_ and T_outlet_ were the inlet and outlet temperatures, respectively. Table 6 shows that, based on the measured values, flow actuation did not improve cooling performance; in fact, it reduced performance by 21%, and the lower temperature was attained due to the low flow rate (accordingly to Equation (4))

**Figure 5:**
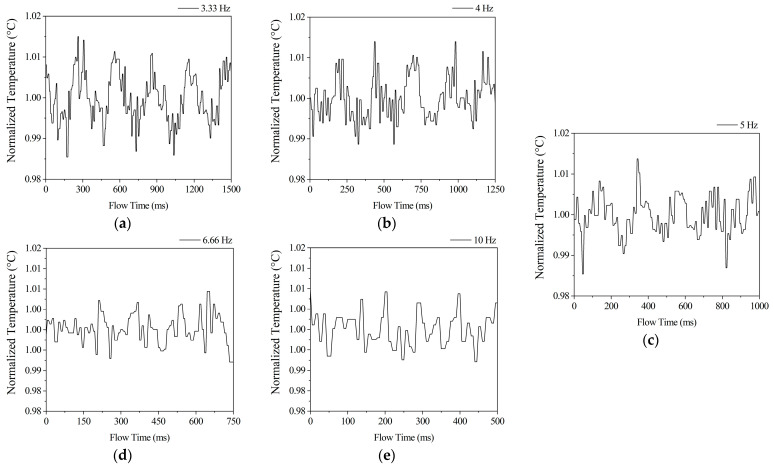
Normalised outlet temperatures at 3.33 Hz (**a**), 4 Hz (**b**), 5 Hz (**c**), 6.66 Hz (**d**), 10 Hz (**e**).

The temperature sensor of type SLF3-1300F overestimated the temperature by 2 °C. Nonetheless, the overprediction remained consistent throughout the measurements. The SLF3-1300F monitoring interval of 5 ms was ideal for depicting temperature fluctuations. Figure 5 depicts normalised outlet temperature samples. Temperature values were also fitted with sinusoidal curves, but the regression values were less than R^2^ = 0.7, so they are not showcased. When Figure 4a and Figure 5a are compared, it becomes clear that the temperature minima occur after the flow rate minima. This phenomenon is also present at 4 and 5 Hz. The amplitudes are damped at 6.6 and 10 Hz.

It is worth noting that the damping in Figure 5d,e cannot be attributed to changes in flow rate or temperature. The reason for this is the fact that the average flow rate difference between 6.6 and 10 Hz is the largest, and the temperature is also significantly different. The amplitude dampening of the temperature can only be attained by increasing the frequency.

The results of the 10 Hz flow might imply that other possible optimums exist at higher frequencies, but constraints of the method prevent this from being investigated. Furthermore, even in the 3.3 Hz scenario, the 10 Hz value was greater. When the frequency is reduced, the temperature and flow rate rise correspondingly.

### 3.2. Numerical Models

The numerical models were created in three stages, with the goal of describing the pulsating flow by developing more and more advanced models. Temperatures from the heat sink surface and fluid were gathered in each stage in the function of time. The velocities were also recorded when the flow was added to the models.

#### 3.2.1. Flow-Less Scenarios

The flow-less scenario demonstrates a classical Newton’s law of cooling representation, with one notable change. The fluid temperature was not constant, it changed accordingly to Equation (4), while the surface temperature of the heat sink followed the lumped heat capacitance model, which can be described as the following (see Equation (5)).

(5) $${\bar{T}}_{S}={\bar{T}}_{F}+\left(T_{0}-{\bar{T}}_{F}\right)\cdot e^{-h\cdot A\cdot {\left(c\cdot \rho \cdot V\right)}^{-1}\cdot \tau }$$

where T_0_ is the initial temperature, h is the convective heat transfer coefficient, A is the wet surface of the heat sink, V is the volume of the fluid in the heat sink and τ is the time. It is worth noting that h was determined with the following Equation (6).

(6) $$h={\dot{Q}}_{c}\cdot {\left({\bar{T}}_{S}-{\bar{T}}_{F}\right)}^{-1}\cdot A^{-1}$$

As the fluid and surface temperatures matched at the beginning, an initial instability developed because the definition (see Equation (6)) of h at the start had to be infinite and should rapidly converge to an h_∞_ value. Models were created for ${\dot{Q}}_{c}$ = 5–20 W with 2.5 W steps with T_0_ = 10 and 20 °C. In each of the 14 cases after 15 s, h converged to h_∞_ = 960.616 ± 0.587 W∙m^−2^∙K^−1^ independently of the temperature or cooling load. Unfortunately, simulation data show that T_S_ and h require an implicit solving method, due to the initial temperature conditions. Based on the flow-less models, h cannot be assumed to be constant during the early period at iso-flux heat conditions; h can only be constant when the fluid temperature is constant. As a response, Equation (7) is proposed, and T_S_ can be determined by being substituted into Equation (5).

(7) $$h=h_{\infty }+2000\cdot e^{-\tau /0.75}$$

Values 2000 and 0.75 were the products of curve fitting of the fourteen h-τ curves.

#### 3.2.2. Steady Flow Scenarios

The steady slow scenarios were examined with the same thermal conditions and at Re 100; 200; 300; 400. These models had the additional flow development domain. With the addition of the inflow, the examined domain gained extra heat, thus both T_S_ and T_F_ converged at a certain value accordingly to Equation (4). The accuracy of these models, compared to the analytical determination method is less than ±0.5 °C.

The result of this set of simulations shows that h has a strong connection (R = 0.999) with the flow rate and is expressed with Equation (8).

(8) h∞=960.616+Re·1.236

This kind of connection is well known in practice. The EN ISO 6946 standard [29] also assumes that the convective heat transfer coefficient depends on the velocity of the flow.

#### 3.2.3. Pulsating Flow Scenarios

In each pulsating flow simulation, 5 W cooling power, 10 °C inlet temperature, and 30 mL∙min^−^^1^ mean pulsating flow rate were established. The sinusoidal wave shape can be divided into cycles, with the initial, middle, and end positions ideally matching. The normalised velocity distribution was evaluated in a radial direction, at three significant cross sections of the analysed region with the presence of sinusoidal inlet velocity.

**Figure 6:**
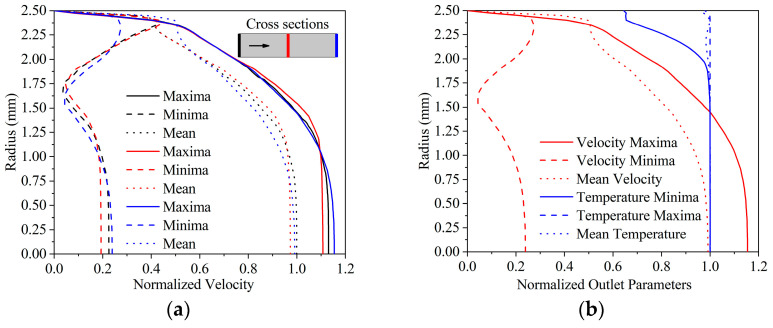
Normalised velocity distribution at different cross sections (**a**), normalised velocity and temperature distribution at the outlet (**b**).

Figure 6a shows that significant differences occur. Figure 6 illustrates the normalised velocity distribution when the cross-section averaged values were at the extremes and the mean magnitude. The velocity profiles at the three cross sections studied had a consistent shape to the corresponding cycle position. Similar traits of the velocity profile have been found in numerical models of blood flow [13]. There are significant changes within the viscous sublayer at the outlet (Figure 6a blue line). The outlet cross-section had the lowest velocity in this near-wall region. The velocity distribution is especially difficult to characterise as a function because the logarithmic layer rises quickly and at about 2.4 mm, a significant break commences the laminar profile. It is also worth noting that the viscous sublayer profile altered just slightly.

In every moment of the cycle, in the centre of the flow domain velocity increased slightly towards the outlet. The increase can be attributed to the development of the velocity profile along its path. It can be also noted that the lowest average flow rate in the numerical model was not zero, the minima were at about 10 mL∙min^−^^1^. The viscous sublayer profile was damping, and the core flow inertia held the velocities at a comparatively (0.2) high magnitude.

The temperature minima profile showed two distinct sections (see Figure 6b), with the temperature difference in the viscous sublayer being minor compared to the wall temperature. The low velocity in the near-wall zone can be attributed to this. A break develops in the profile further away from the wall and the temperature rises considerably along with the velocity. Since the water cannot cool down more if the velocity is high. At lower velocities, the temperature distribution becomes homogenous, resulting in a minimum temperature difference in the near-wall zone. The temperature distribution is unaffected by the velocity drop of roughly at a 1.6 mm radius in the velocity minima profile. Since the velocity remained at this magnitude for a brief time.

Table 7 displays the averaged parameters of the final three seconds (600 time steps) of the pulsating flow simulations. Three seconds was the cycle’s least common multiple. The temperature and flow rates were both quite close to one another, with variances of less than 1%. The most favourable result was obtained at 3.3 Hz, with an increase of 0.63%. However, it should be emphasised that the cooling power was just 5 W, therefore no enhancement was obtained. Approximately 0.4 W or nearly 10% of the input cooling power, was lost in the process.

**Figure 7:**
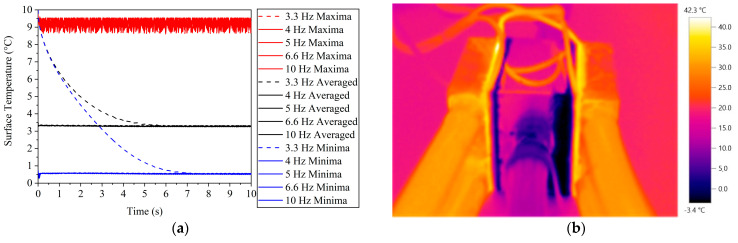
Surface temperatures of the heat sink with numerical method (**a**) and with Testo 881 thermal camera (**b**).

The surface temperatures of the heat sink were measured to identify a reason for the loss of cooling power. Figure 7a shows that the temperature maxima were less than 10 °C lower than the inflow temperature, which would be the ideal circumstance. Because there was convective thermal resistance on the heat sink’s exterior surface, it was also cooled. Thus, the effectiveness of cooling was determined by the quality of the heat transfer coefficient. As can be seen, the 3.3 Hz had a significant lag throughout the initial period, and it finally converged to a stable value after the seventh second.

This low temperature (T_cold_) can be also seen in Figure 7b. The temperature change in the bulk of the heat sink was small due to the relatively high thermal conductivity of aluminium at the heat source. It can be also noted that the cooling circuit was effective since it kept the T_warm_ low. Because of the reflecting properties of the metal surface, precise quantities cannot be established.

#### 3.2.4. Verification

The measured values and the simulation results were compared to examine the viability of the chosen numerical model. The flow-less simulation was not examined, due to the intense icing and the lack of phase change in the model.

The steady flow model was compared with the parameters listed in Table 8 and with the modelled outcomes. Because the outlet and surface temperatures were not established in the simulation, it was possible to check the values and apply them for verification. Although the relative change in surface temperature was significant, the absolute difference is less than a quarter degree of Celsius, indicating a satisfactory agreement.

It was difficult to compare pulsing flow rates. The experimental flows produced peak flows as a result of the actuation, resulting in insignificant negative flow records, whereas models showed no backflows. Because of these features, the comparison must be conducted independently or in specific relation to the flow rate. As a result, the ratio of temperature difference to mean flow rate was examined at various frequencies.

It can be observed that as the frequency rises, the disparity between the two types of results grows (see Table 9). The model has a strong consistent ratio that declines slightly at 10 Hz. While the measured results reveal significant variations at higher frequencies, knowing that the differences allow for a more appropriate prediction assessment.

## 4. Discussion

The Nusselt number (Nu) was used to evaluate heat transfer performance. Nu is expressed in Equation (9) as a function of the thermal conductivity of water (λ = 0.6 W∙m^−^^1^∙K^−^^1^) and that of the previously defined D and h. This equation can be combined with Equation (6).

(9) $$\mathrm{Nu}=h\cdot D\cdot \lambda^{-1}={\dot{Q}}_{c}\cdot D\cdot {\left(\left({\bar{T}}_{S}-{\bar{T}}_{F}\right)\cdot A\cdot \lambda \right)}^{-1}$$

While numerical results can easily provide the avenged temperature difference, measuring requires a different approach.

Overtemperature difference, which is the difference between the heat sink surface and the average flow temperatures. The average flow temperature can be attained by volume averaging the fluid bulk temperatures (T_F_). With this method, the volume-averaged overtemperature difference (ΔTa) can be expressed as the following:

(10) $$\Delta \mathrm{Ta}={\bar{T}}_{S}-{\bar{T}}_{F}$$

Although the ΔTa is the most accepted approach for determining h, it is primarily feasible by modelling the flow. Because the temperature distribution of the whole volume of fluid is problematic, verifying the T_F_ is challenging. When the initial and treated temperatures are measured and averaged, the process is simplified. When the temperatures at the inlet and outlet cross-sections are averaged, an averaged over temperature (ΔTb) is calculated:

(11) $$\Delta \mathrm{Tb}={\bar{T}}_{S}-\left(T_{\mathrm{outlet}}+T_{\mathrm{inlet}}\right)\cdot 0.5$$

Inlet temperature difference (ΔTc, Equation (11)) can be applied when the fluid temperature change is small, according to R. Webb [30].

(12) $$\Delta \mathrm{Tc}={\bar{T}}_{S}-T_{\mathrm{inlet}}$$

This approach implies that the surface has no influence on the flow, consequently, the temperature change is not possible. Fox external flows, these strategies are most commonly used [31,32]. Webb [30] also said that when the flow temperature varies significantly, logarithmic mean temperature difference (ΔTd) can be employed, which may be expressed using the equations below (Equation (13)).

(13) $$\begin{array}{c} \Delta T_{1}=T_{\mathrm{outlet}}-T_{S\;\min}\\ \Delta T_{0}=T_{\mathrm{inlet}}-T_{S\;\max}\\ \Delta \mathrm{Td}=\left(\Delta T_{1}-\Delta T_{0}\right)\cdot \ln{\left(\Delta T_{1}\cdot \Delta T_{0}{}^{-1}\right)}^{-1} \end{array}$$

The approach is commonly used in the design of heat exchangers. It was discovered that the temperature distribution on the heat sink was not even in the simulation. The ΔTd is distorted by the peaked temperature records at the boundaries.

Three of the last-mentioned temperature difference calculation method (ΔTb, ΔTc, ΔTd) overpredicts the first method (ΔTa) (see Table 10), thus making it impossible to assess Nu solely by measurements. It should be emphasised that heat transfer measurements should be done simultaneously with numerical models.

The heat transfer process should be investigated not only with the nondimensional number that corresponds to this process (Nu), but also with the pulsations. The Wormesly number (Wo) is extensively used to assess pulsating flows [33] (see Equation (14)).

(14) $$\mathrm{Wo}=0.5\cdot D\cdot {\left(f\cdot \rho \cdot \mu^{-1}\right)}^{0.5}\;$$

However, because this value does not contain the flow rate, the Strouhal number (St) (see Equation (15)) may be preferable in these instances or should be stated at which Re was the experiment conducted.

(15) $$\mathrm{St}=D\cdot f\cdot v^{-1}\;$$

**Figure 8:**
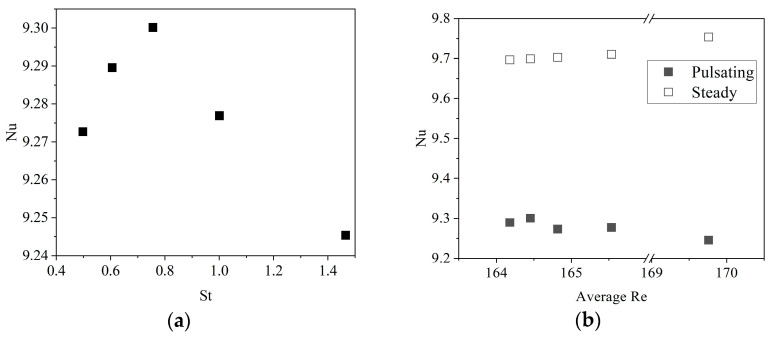
Nusselt numbers at different Strouhal numbers (**a**) and at averaged Re (**b**).

In Figure 8, the Nu can be seen in the function of St. The Nusselt number peak can be seen at 6.6 Hz. The magnitude of the increase corresponds to the change in cooling power.

The presence of the pulsations in flow does not enhance the heat transfer. However, in the examined range, the various frequencies had a slight influence on the Nu. This kind of relation between the frequency and Nu was shown in the work of Poh et al. [34] at Re 100. The Nu difference between steady and pulsating flow can only be assessed in terms of the average Re. Figure 8b illustrates a 4.3–5.5% decrease with the pulsations when compared to its constant flow version using Equation (8).

### 4.1. Limitations

The limitation of the work as follows:

- The solenoid valve actuation does not allow to have pulses higher than 10 Hz.
- The flow rate is limited to 60 mL∙min^−^^1^ due to the limit of the measuring apparatus.
- The simulation had no radiation model since the water was transparent and the heat sink was opaque.
- Phase change was not modelled, the temperature was above 0 °C at 1 atm pressure.

### 4.2. Future Research Directions

The objective for the future is to improve the experimental side by making measurements in larger frequency ranges and at higher flow rates while remaining in the laminar area. The use of a shock damper is also essential to reduce the distortion of the flow rate minima. More simulation adjustments are required to eliminate the minimum flow issue.

## 5. Conclusions

Pulsating flows were examined both experimentally and with a combination of large-eddy simulation and thermal model. Scenarios were carried out at 3.3; 4; 5; 6; and 10 Hz in the laminar regime around Re = 127.

For the iso-flux condition, the lumped heat capacitance model was evaluated. Correction is required based on the numerical result since the convective heat transfer coefficient is not constant, but rather develops over time.

A slight peak was found at Nu = 9.3 at St = 0.756, yet in the measured range, no considerable enhancement was found.

Nu was lower when compared to constant flow scenarios at the corresponding average Re.

The results of both experiments and models show good agreement. With simulation, new optimum ranges can be found and verified by the showcased experimental method.

## Figures and Tables

**Table 1:** Measured parameters.

Type	Parameter	Accuracy
KIMO KTH-300 with KRV-200	T_inlet_; T_outlet_	0.1 °C
NTC Temperature Sensor	T_cold_; T_warm_	
Testo 881		
Sensirion SLF3-1300F	$\dot{V}$; T_outlet_	0.05 mL∙min^−^^1^ and 0.02 mL∙min^−^^1^∙°C^−^^1^
DT321B	U	0.01 V
REV Ritter EMT717ACTL	Ṗ	1 W

**Table 2:** Grid convergence index calculation.

Mesh Density	Element Number	y^+^	Outlet Temperature (K)	GCI
coarse	238,707	0.4	279.994	−0.032%
medium	888,190	0.2	280.066	-
Fine	3,480,884	0.1	280.102	−0.016%

**Table 3:** Parameters of the curve fitting.

Frequency	3.3 Hz	4 Hz	5 Hz	6.6 Hz	10 Hz
f (ms)	3.362 ± 0.002	4.033 ± 0.004	5.029 ± 0.007	6.702 ± 0.014	9.967 ± 0.023
φ (ms)	243.379 ± 1.999	161.608 ± 1.573	124.747 ± 1.065	42.648 ± 0.771	−25.083 ± 0.772
$\bar{\dot{V}}$ (mL∙min^−^^1^)	26.399 ± 0.959	24.052 ± 0.93	20.446 ± 0.794	19.665 ± 0.666	31.196 ± 0.817
β_V_ (mL∙min^−^^1^)	50.437 ± 1.337	54.476 ± 1.31	57.377 ± 1.123	56.074 ± 0.936	49.054 ± 1.155
R^2^	0.826	0.852	0.897	0.923	0.807

**Table 4:** Measured flow rates.

Frequency	3.3 Hz	4 Hz	5 Hz	6.6 Hz	10 Hz
Flow rate (mL∙min^−^^1^)	26.682 ± 39.47	24.318 ± 41.593	20.185 ± 42.249	19.788 ± 40.996	30.936 ± 32.201
Relative Flow Rate Change	32%	20%	-	−2%	53%

**Table 5:** Measured temperatures.

Frequency	3.3 Hz	4 Hz	5 Hz	6.6 Hz	10 Hz
Inlet Temperature (°C)	11.652 ± 0.019	11.627 ± 0.006	11.635 ± 0.021	11.652 ± 0.019	11.614 ± 0.013
Outlet Temperature (°C)	8.782 ± 0.02	9.203 ± 0.012	8.51 ± 0.031	8.782 ± 0.02	9.143 ± 0.042
Temperature Difference (°C)	2.87	2.424	3.125	2.87	2.471
Relative Change	−8%	−22%	-	−8%	−21%

**Table 6:** Calculated cooling power from experimental data.

Frequency	3.3 Hz	4 Hz	5 Hz	6.6 Hz	10 Hz
Cooling Power (W)	5.325	4.099	4.386	3.949	5.315
Relative Change	21%	−7%	-	−10%	21%

**Table 7:** Modelled temperatures at 10 °C inlet temperature.

Frequency	3.3 Hz	4 Hz	5 Hz	6.6 Hz	10 Hz
Outlet temperature (°C)	8.29 ± 0.99	8.29 ± 0.96	8.30 ± 0.97	8.31 ± 0.96	8.35 ± 0.92
Temperature difference (°C)	1.71	1.71	1.70	1.69	1.65
Relative temperature change	0.41%	0.23%	-	−0.70%	0.00%
Flow rate (mL∙min^−1^)	39.021 ± 20.25	38.87 ± 20.06	38.94 ± 19.82	39.19 ± 19.66	40.19 ± 19.37
Relative flow rate change	0.22%	−0.17%	-	0.66%	3.23%
Cooling power (W)	4.64	4.62	4.61	4.61	4.62
Relative cooling power change	0.63%	0.07%	-	−0.05%	0.20%

**Table 8:** Comparison of the steady flow.

Parameter	Measured	Modelled	Relative Change
Inlet temperature (°C)	20.4		
Flow rate (mL∙min^−^^1^)	46.9		
Cooling power (W)	20		
Outlet temperature (°C)	16.5	16.46	0.24%
Surface temperature (°C)	0.5	0.36	28%

**Table 9:** Temperature difference and flow rate ratios.

Frequency	3.3 Hz	4 Hz	5 Hz	6.6 Hz	10 Hz
Experimental (°C∙min∙mL^−^^1^)	0.025	0.024	0.037	0.034	0.019
Modelled (°C∙min∙mL^−^^1^)	0.027	0.027	0.027	0.027	0.026
Relative flow rate change	8.00%	12.50%	−27.03%	−20.59%	36.84%

**Table 10:** Temperature differences with different calculation methods.

Method	ΔTa (Equation (10))	ΔTb (Equation (11))	ΔTc (Equation (12))	ΔTd (Equation (13))
Averaged Temperature Difference (°C)	4.399	5.862	6.714	7.439

## Data Availability

Not applicable.
